# Reticulon-1C Involvement in Muscle Regeneration

**DOI:** 10.3390/metabo11120855

**Published:** 2021-12-08

**Authors:** Federica Rossin, Elena Avitabile, Giorgia Catarinella, Ersilia Fornetti, Stefano Testa, Serafina Oliverio, Cesare Gargioli, Stefano Cannata, Lucia Latella, Federica Di Sano

**Affiliations:** 1 Department of Biology, University of Rome ‘Tor Vergata’, 00133 Rome, Italy; elena.avitabile@tim.it (E.A.); ersiforn@hotmail.it (E.F.); stefanotesta87@hotmail.it (S.T.); oliverio@bio.uniroma2.it (S.O.); cesare.gargioli@uniroma2.it (C.G.); cannata@uniroma2.it (S.C.); federica.di.sano@uniroma2.it (F.D.S.); 2 Epigenetics and Regenerative Medicine, IRCCS Fondazione Santa Lucia, 00179 Rome, Italy; giorgia.cat.94@gmail.com (G.C.); l.latella@hsantalucia.it (L.L.); 3 DAHFMO, Unit of Histology and Medical Embryology, Sapienza University of Rome, 00185 Rome, Italy; 4 Institute of Translational Pharmacology, National Research Council of Italy, 00133 Rome, Italy

**Keywords:** RTN-1C, muscle differentiation, Duchenne muscular dystrophy, UPR

## Abstract

Skeletal muscle is a very dynamic and plastic tissue, being essential for posture, locomotion and respiratory movement. Muscle atrophy or genetic muscle disorders, such as muscular dystrophies, are characterized by myofiber degeneration and replacement with fibrotic tissue. Recent studies suggest that changes in muscle metabolism such as mitochondrial dysfunction and dysregulation of intracellular Ca^2+^ homeostasis are implicated in many adverse conditions affecting skeletal muscle. Accumulating evidence also suggests that ER stress may play an important part in the pathogenesis of inflammatory myopathies and genetic muscle disorders. Among the different known proteins regulating ER structure and function, we focused on RTN-1C, a member of the reticulon proteins family localized on the ER membrane. We previously demonstrated that RTN-1C expression modulates cytosolic calcium concentration and ER stress pathway. Moreover, we recently reported a role for the reticulon protein in autophagy regulation. In this study, we found that muscle differentiation process positively correlates with RTN-1C expression and UPR pathway up-regulation during myogenesis. To better characterize the role of the reticulon protein alongside myogenic and muscle regenerative processes, we performed in vivo experiments using either a model of muscle injury or a photogenic model for Duchenne muscular dystrophy. The obtained results revealed RTN-1C up-regulation in mice undergoing active regeneration and localization in the injured myofibers. The presented results strongly suggested that RTN-1C, as a protein involved in key aspects of muscle metabolism, may represent a new target to promote muscle regeneration and repair upon injury.

## 1. Introduction

Myogenic differentiation is an essential process of muscle development depending on the activity of different specific cells and environmental signals [1,2]. While the number of skeletal muscle fibers remains constant after achieving adulthood, skeletal muscle metabolism and mass are influenced by various factors such as diet, genetics, hormones, growth factors, and mechanical stimuli [3].

Loss of skeletal muscle mass is associated with a wide array of disease states such as aging, cancer, HIV, chronic heart failure, burn injury, etc. [4]. In addition, myofiber degeneration is a common feature of genetic muscle disorders, such as muscular dystrophies, characterized by the myofibers’ replacement with fibrous and fat tissue [5]. Skeletal muscle contains an extremely extensive network of specialized endoplasmic reticulum (ER), namely sarcoplasmic reticulum. ER functional disturbance causes ER stress and leads to the accumulation of unfolded or misfolded proteins, thereby triggering the unfolded protein response (UPR) to alleviate cellular stress and re-establish homeostasis [6,7].

ER stress and the UPR pathways may regulate not only skeletal muscle adaptation in response to physiological stimuli, but also their formation during embryonic development and regeneration upon damage. Indeed, recent studies suggest that a physiological mild ER stress, which occurs during muscle cell differentiation, improves myogenic differentiation efficiency [8,9]. On the other hand, evidence indicates that ER stress and UPR are activated in various types of myopathies as well. Although chronic ER stress can cause pathological alterations contributing to skeletal muscle wasting, it is now emerging that ER stress can have distinct outcomes in different disorders and opposite effects in relation to disease stages [10].

Existing evidence also links muscular dystrophies with mitochondrial and metabolic dysfunction. Indeed, it is now clear that energetics and mitochondrial dynamics are severely affected in muscle disorders and multiple metabolic adaptations are induced to maintain energy homeostasis in dystrophic muscle [11].

In this regard, the reticulon 1 protein (RTN-1C) has been demonstrated to be a key component of the ER compartment and a functional molecule in the induction of ER stress. RTN-1C is a member of the reticulon family, ER-associated proteins that play important role in bending and shaping the ER membrane, in trafficking of material from the ER to the Golgi apparatus, and in apoptosis [12]. Our previous works demonstrated that RTN-1C regulates ER membranes morphology, cytosolic calcium levels, and UPR induction [13,14,15]. Specifically, we found that RTN-1C is a key component of the MAM compartment. As such, it regulates mitochondrial function and Ca^2+^ homeostasis, thus providing a possible mechanism by which this structural ER protein modulates the cellular metabolism. Recently, we also demonstrated that RTN-1C is involved in the regulation of autophagy [16]. Specifically, changes in RTN-1C expression levels reflect autophagy modulation affecting autophagosomes formation. Interestingly, autophagy is emerging as an important process driving muscle differentiation and regeneration [17,18,19]. Indeed, basal autophagy seems to be critical for maintenance of skeletal muscle mass as well as its function and metabolism.

Based on these findings, we here studied the role of RTN-1C in myogenic and muscle regenerative processes. We provided evidence that RTN-1C expression correlates with UPR pathway up-regulation and myogenic differentiation. Moreover, by using an in vivo model of Duchenne muscular dystrophy, we observed that RTN-1C modulation is also associated with myogenic and muscle regenerative processes in a pathogenic context.

## 2. Results

**Figure 1:**
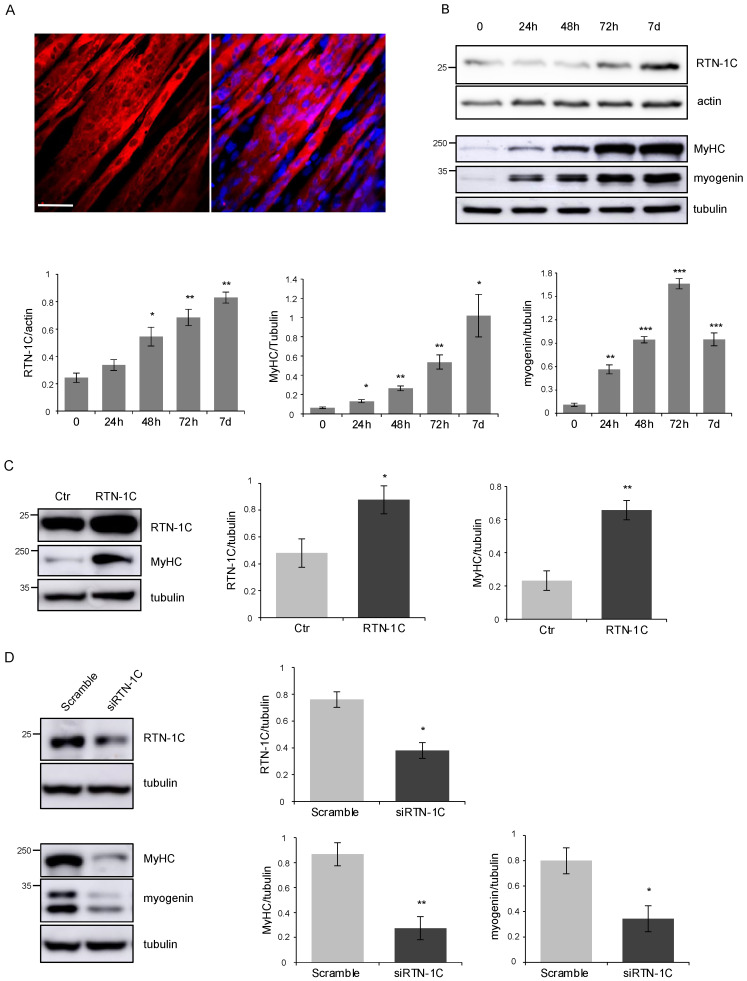
(**A**) MyHC immunofluorescence images of C2C12 myotubes upon myogenic differentiation for 7 days. Scale bar: 20 μm. (**B**) Representative western blot and densitometric analysis of RTN-1C in C2C12 cells upon differentiation induction, by culture in DM for the indicated times. MyHC and myogenin were used as markers of differentiation process. Actin and tubulin were used as loading control. (*n* = 3; means ± SEM; * *p* < 0.05; ** *p* < 0.01; *** *p* < 0.001). (**C**) Representative western blot and densitometric analysis of RTN-1C and MyHC in C2C12 cells after transfection with RTN-1C vector. Ctr represents cells transfected with the empty vector. Tubulin was used as loading control. (*n* = 3; means ± SEM; * *p* < 0.05; ** *p* < 0.01). (**D**) Representative western blot and densitometric analysis of RTN-1C, MyHC and myogenin in C2C12 cells silenced for RTN-1C and cultured in DM for 72 h to induce differentiation. Scramble oligos were used as control. Tubulin was used as loading control. (*n* = 3; means ± SEM; * *p* < 0.05; ** *p* < 0.01).

To investigate RTN-1C’s role in skeletal muscle differentiation, we used C2C12 myoblasts, a well-established cellular model, to study myogenesis. C2C12 can be cultivated in proliferative conditions when cultured in growth medium (GM) and undergo myogenic differentiation once shifted in differentiation medium (DM), forming multinucleated differentiated myotubes as shown in Figure 1A. We monitored the expression of RTN-1C during the differentiation process by western blotting (Figure 1B). In particular, we followed the myogenic differentiation at different time points after DM induction. We found that the protein level increased after 48 h of DM exposure and correlated with the expression of myogenin, an early myogenic regulator. Interestingly, RTN-1C levels kept increasing at later stages of differentiation (7 days), as highlighted by the expression of myosin heavy chain (MyHC), a muscle-specific late marker. These results suggested the involvement of RTN-1C in the muscle differentiation process.

To study the effect of RTN-1C modulation along the differentiation process, we either over-expressed or silenced the protein in C2C12 cells and analyzed by western blotting the expression of different myogenic markers. Figure 1C shows that the ectopic over-expression of RTN-1C for 72 h was able to increase MyHC protein levels. Accordingly, silencing the protein drastically affected the expression of both myogenin and MyHC (Figure 1D), thus proposing RTN-1C as a positive regulator of muscle differentiation.

**Figure 2:**
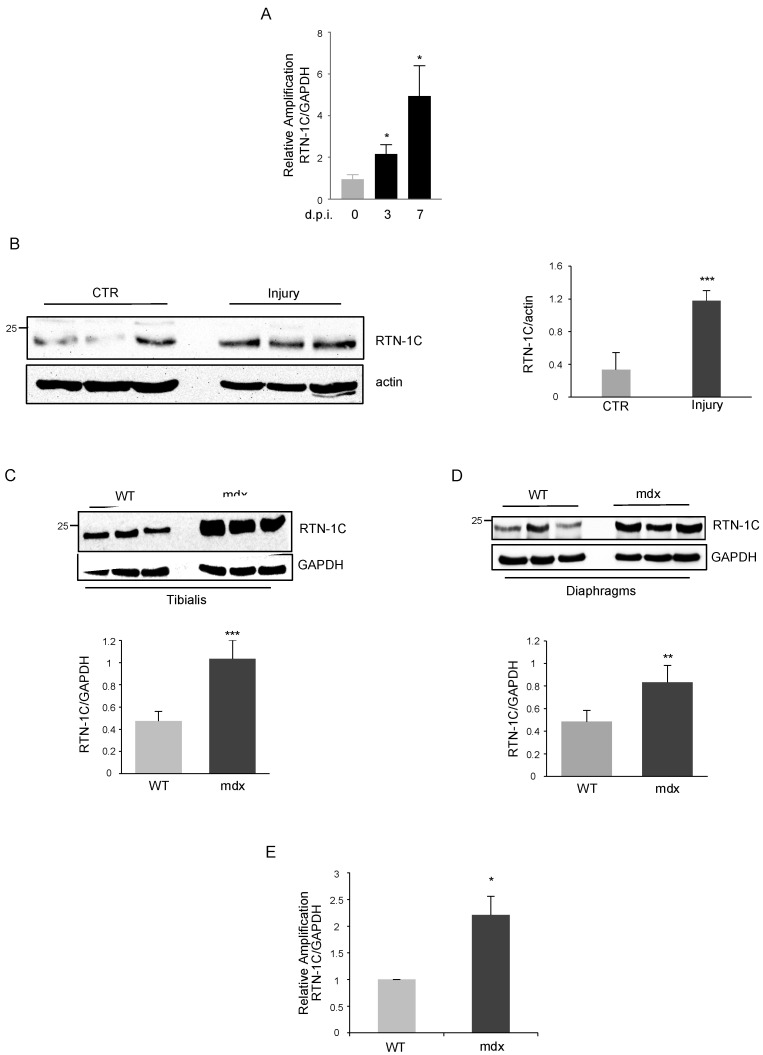
(**A**) RTN-1C mRNA expression in anterior tibialis from WT and injured WT mice 3 and 7 days post injury (d.p.i.), measured by qPCR and normalized for GAPDH. (*n* = 3; means ± SEM; * *p* < 0.05). (**B**) Representative western blot and densitometric analysis of RTN-1C in tibialis muscles, from WT mice, subjected to injury and analyzed 3 d.p.i. Actin was used as loading control. (*n* = 8; means ± SEM; *** *p* < 0.001). (**C**,**D**) Representative western blot and densitometric analysis of RTN-1C in protein extracts from tibialis and diaphragm muscles of WT and mdx mice at 3 months of age. GAPDH was used as loading control. (*n* = 8; means ± SEM; ** *p* < 0.01; *** *p* < 0.001). (**E**) RTN-1C mRNA levels, quantified by qPCR and normalized for GAPDH, in tibialis muscles from WT and mdx mice at 3 months of age. (*n* = 8; means ± SEM; * *p* < 0.05).

Considering skeletal muscle’s ability to regenerate in response to injury, we investigated on the role of RTN-1C during the regenerative processes in vivo. Muscle regeneration was induced by muscle freezing of anterior tibialis in wild type (WT) mice and analysed for the expression of RTN-1C after 3 and 7 days post injury (d.p.i.). RTN-1C expression in injured anterior tibialis significantly increased at transcriptional (Figure 2A) and translational (Figure 2B) levels compared with uninjured muscles, suggesting a role of RTN-1C during the regenerative process in vivo.

Prompted by this evidence, we investigated whether RTN-1C was involved not only in physiological regeneration, but also in disease-associated regenerative processes occurring in Duchenne Muscular Dystrophy (DMD). To this aim, we used mdx mice, the most common mouse model for DMD, characterized by the presence of a premature stop codon in exon 23 of the dystrophin gene. In mdx mice, muscle fibers became necrotic, initiated inflammatory processes, and progressed through cycles of degeneration and regeneration, starting at 2 weeks of age, peaking at 12 months, and continuing for the remaining lifetime of the mouse. Remarkably, the analysis of RTN-1C levels underlined a higher amount of the protein in both tibialis anterior and diaphragms of mdx mice with respect to the age-matched WT mice (Figure 2C,D). Accordingly, in tibialis of dystrophic mice RTN-1C mRNA levels were increased (Figure 2E).

**Figure 3:**
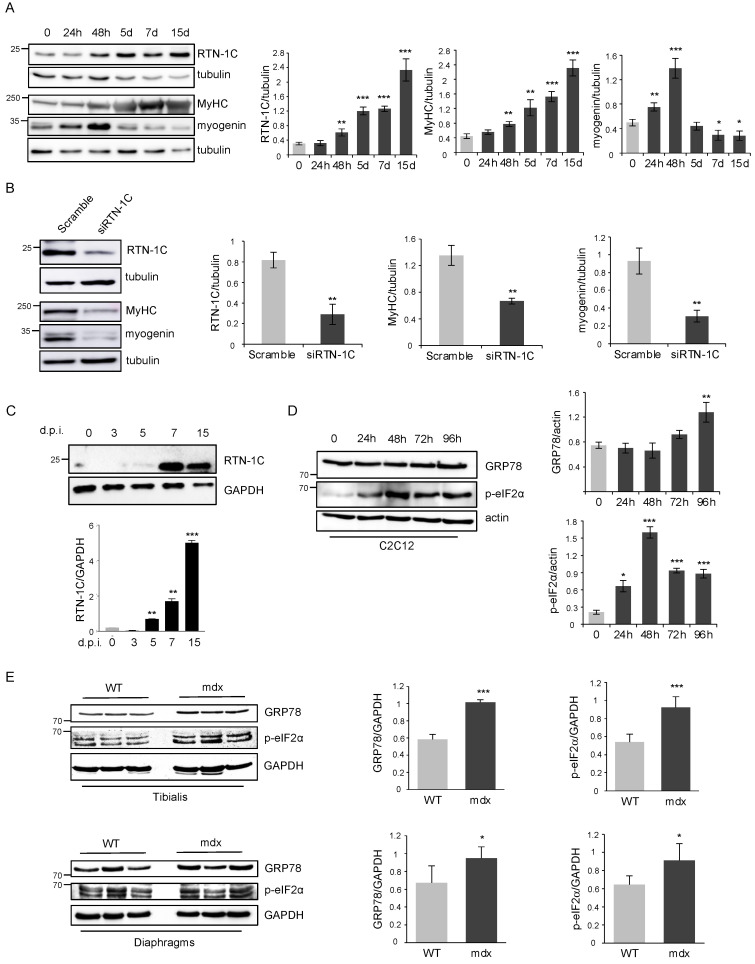
(**A**) Representative western blot and densitometric analysis of RTN-1C in satellite cells, isolated from WT mice tibialis, exposed to differentiation medium for different time points. MyHC and myogenin were used as markers of the differentiation process. Tubulin was used as loading control. (*n* = 3; means ± SEM; * *p* < 0.05; ** *p* < 0.01; *** *p* < 0.001). (**B**) Representative western blot and densitometric analysis of RTN-1C, MyHC, and myogenin in satellite cells, isolated from WT mice, silenced for RTN-1C (siRTN-1C) and cultured in DM for 72 h to induce differentiation. Scramble oligos were used as control. Tubulin was used as loading control. (*n* = 3; means ± SEM; ** *p* < 0.01). (**C**) Representative western blot and densitometric analysis of RTN-1C expression in FACS-sorted satellite cells, analyzed after isolation in WT mice and injured mice at 3, 5, 7, and 15 days post injury (d.p.i.). (*n* = 3; means ± SEM; ** *p* < 0.01; *** *p* < 0.001). (**D**) Representative western blot and densitometric analysis of GRP78 and p-eIF2α in C2C12 cells upon differentiation induction, by culture in DM, for the indicated times. Actin was used as loading control. (*n* = 3; means ± SEM; * *p* < 0.05; ** *p* < 0.01; *** *p* < 0.001). (**E**) Representative western blot and densitometric analysis of GRP78 and p-eIF2α in protein extracts from tibialis and diaphragm muscles of WT and mdx mice at 3 months of age. GAPDH was used as loading control. (*n* = 8; means ± SEM; * *p* < 0.05; *** *p* < 0.001).

During muscle regeneration, different cell types take part of the repair process. Among those, we can include muscle satellite cells, macrophages, and fibro-adipogenic precursors [20]. In particular, we wanted to evaluate the expression of RTN-1C in satellite cells, the skeletal muscle adult stem cell compartment that regulates regenerative processes in adulthood. To this end, we performed ex-vivo cultures of primary satellite cells isolated from mouse tibialis muscles then exposed to differentiation medium for the indicated time (Figure 3A). The expression of the early differentiation marker myogenin increased in the first 5 days while the RTN-1C protein level correlated to the late marker MyHC, rising after day 5 up to 15, corresponding to terminally differentiated myotubes. Hence, the silencing of RTN-1C affected myogenin and MyHC protein expression (Figure 3B), further supporting the involvement of the RTN-1C protein in the myogenic differentiation process.

To trace RTC-1C expression in satellite cells in vivo, we analyzed its expression in freshly-isolated cells at different time points after muscle injury and analyzed right after isolation. Upon muscle injury, satellite cells exited from the quiescent state and became activated. After a proliferative expansion, they eventually underwent myogenic differentiation to repair damaged muscle [21,22,23]. We analyzed early (3 and 5 d.p.i.) and late (7 and 15 d.p.i.) stages of the regenerative process, corresponding to expansion phase and commitment toward muscle differentiation, respectively. As shown in Figure 3C, we detected a progressive increase of RTN-1C in satellite cells, reaching the highest levels at 15 days p.i. These data provided a direct demonstration suggesting that RTN-1C displays a role in muscle differentiation during physiological muscle regeneration in vivo.

We previously demonstrated that RTN-1C plays an important role in endoplasmic reticulum (ER) stress induction and the UPR pathway [13,14]; interestingly, both processes have recently been involved in myogenic differentiation. In particular, it has been demonstrated that ER stress is induced during myogenesis to improve myotubes formation [9].

In line with these findings, we observed an increase of ER stress-related genes such as GRP78 and the phosphorylated form of eIF2α that parallels RTN-1C upregulation in C2C12 (Figure 3D). These data were also confirmed in satellite cells (data not shown), suggesting that the RTN-1C induction could be functional to generate ER stress during myogenesis, favoring the formation of mature myotubes.

According to this evidence, we investigated whether RTN-1C could affect ER stress response during DMD pathogenesis. We unveiled a positive correlation among RTN-1C-increased expression in dystrophic tibialis and diaphragm and enhanced GRP78 and phosphorylated eIF2α protein levels (Figure 3E).

## 3. Discussion

In this study, we investigated the involvement of the reticulon protein 1C (RTN-1C) in skeletal muscle differentiation. We previously demonstrated that RTN-1C has a key role in different cellular processes associated to changes in ER homeostasis and function [14]. Skeletal muscle contains an extensive network of endoplasmic reticulum, which plays an important role in the regulation of proteostasis and calcium homeostasis. Different environmental and genetic factors may result in ER function deregulation and ultimately lead to ER stress. To alleviate the stress and restore homeostasis, the ER activates a signaling network called the unfolded protein response (UPR). Many studies recently explored the role of UPR pathways in skeletal muscle, suggesting pivotal roles in muscle stem cell homeostasis, myogenic differentiation, and regeneration of injured skeletal muscle [6]. Autophagy is another intracellular mechanism maintaining healthy muscle homeostasis and physiology. It has been demonstrated that it is essential not only to sustain the energy needs and to control muscle mass, but it is also important for muscle regeneration and for the prevention of different muscle disorders [17,18,19,24].

Another important aspect of skeletal muscle metabolism and physiology is mitochondrial dynamics, which affect the signaling pathways regulating muscle mass. In this regard, we demonstrated that RTN-1C induces morphological and functional changes of mitochondria through the modulation of ER–mitochondria cross-talk [15].

Moreover, we recently highlighted a new role for RTN-1C protein as a novel emerging regulator of autophagic processes [16]. Taken together, all this evidence prompted us to investigate the possible role of RTN-1C in skeletal muscle regeneration by the use of in vitro, ex-vivo, and in vivo experimental approaches. To this end, we first analyzed reticulon expression during the differentiation process of C2C12 myoblasts into multinucleated myotubes. As expected, in these cells, we observed a time-dependent up-regulation of different typical markers of myogenesis but, interestingly, we also found a significant enhancement of RTN-1C expression correlating with the differentiation degree. When we modulated the expression of the reticulon protein by overexpressing or silencing experiments, we were able to induce or inhibit, respectively, both myogenin and MyHC expression. Taken together, these data suggested a likely positive role of RTN-1C in muscle differentiation process.

To further support this notion, we decided to explore the involvement of the reticulon protein in myogenic and muscle regenerative processes in vivo. We investigated RTN-1C modulation after muscle freeze injury, a widely used model system to study the events involved in muscle regeneration. The increased expression of RTN-1C in the damaged tissue possibly suggests that the induction of the protein after muscle injury advances the muscle regeneration processes.

To determine the contribution of RTN-1C, we analyzed RTN-1C levels in a mouse model of Duchenne muscular dystrophy (mdx), which is characterized by degeneration and regeneration of myofibers upon satellite cells activation. We demonstrated that mRNA and protein levels of the reticulon protein are up-regulated in mdx mice with respect to wild-type mice.

The skeletal muscle has an intrinsic capacity to regenerate tissue damage deriving from physiological or pathological injuries. This ability is widely accomplished by undifferentiated skeletal muscle precursor cells, namely satellite cells, located in the niche between the muscle fiber plasmalemma and the basement membrane [25]. Satellite cells are in a quiescent state but can be activated upon muscle injury. Together with other cell types such as macrophages and fibro-adipocyte precursors, satellite cells orchestrate muscle regeneration to repair damaged tissue.

Thus, we investigated the expression of RTN-1C in primary satellite cells, isolated from mouse hind limbs muscles, and which underwent muscle differentiation. Here, again, we observed the up-regulation of the reticulon protein along with the early and late myogenic markers, while the specific silencing of RTN-1C showed an opposite effect, further supporting the involvement of the protein in the muscle differentiation process.

The results obtained so far are not surprising considering the fact that among the different roles of reticulon proteins they have been originally indicated as markers of different cellular differentiations [26]. Moreover, other RTN genes showed high and specific expression levels in smooth and skeletal muscle tissue [27].

As mentioned above, the RTN-1C protein is able to induce ER stress and UPR pathways [13,14] that are known to be activated in skeletal muscle in different conditions and may have important roles in the pathogenesis of inflammatory myopathies and genetic muscle disorders [6]. In particular, ER stress and the UPR response are activated in various types of myopathies including DMD. ER stress markers, such as GRP78, PERK, eIF2α, and caspase12 are increased in the dystrophic muscle of mdx mice as well as in muscle biopsies from DMD patients, suggesting that Dystrophin deficiency disrupts ER homeostasis in DMD affected skeletal muscle [8]. According to the increase of RTN-1C expression, we found that both C2C12 and satellite cells showed the up-regulation of ER stress-related genes such as GRP78 and the phosphorylated form of eIF2α. Moreover, we revealed enhanced GRP78 and phosphorylated eIF2αprotein levels in different tissues of mdx mice compared with controls.

Taken together, these results suggested that the RTN-1C induction could be functional to generate ER stress during myogenesis favoring skeletal muscle differentiation.

As previously mentioned, the preservation of a normal structure and function of skeletal muscle fibers depends on several factors such as cell-cell communication, vesicles-based delivery system, and the maintaining of calcium homeostasis. Considering the involvement of reticulons in ER- vesicle trafficking and the ability of RTN-1C to modulate intracellular calcium concentration and mitochondrial dymanics [14,15], we reason that this protein may have a prominent role in the regeneration of skeletal muscle fibers. In this context, RTN-1C might be recognized as a new target to design more appropriate therapeutic approaches to improve muscle regeneration and to counteract muscle diseases.

## 4. Materials and Methods

### 4.1. Cells

C2C12 cells (less than 10 passage) were cultured in Dulbecco’s modified Eagle’s medium (Lonza, Switzerland) supplemented with 10% fetal bovine serum, 100 μg/mL streptomycin, and 100 units/mL penicillin, at 37 °C and 5% CO_2_ in a humidified atmosphere (GM). To induce differentiation, cells were cultured in Dulbecco’s modified Eagle’s medium (Lonza) supplemented with 2% horse serum (DM).

To silence RTN-1C, cells were transfected with 10-nM siRNA specific for mouse RTN-1C (SR420071, Origene, USA) for 72 h. Scramble siRNA, which does not target any known genes from any species, was used as negative control.

To over-express RTN-1C, human RTN-1C cloned in pCDNA 3.1 Zeo(þ) vector was used [28].

Transfections were performed using Lipofectamine 2000 (Invitrogen, UK) according to the manufacturer’s instructions.

### 4.2. Mice

Three-month-old male wild-type C57BL/6 (WT) mice or C57BL/6ScSn-Dmdmdx/J (mdx) mice purchased from Jackson Laboratories were used. Mice were maintained under regular housing conditions with standard access to food and drink in a pathogen-free facility. All the procedures in mice were approved by the local Ethics Committee for Animal Welfare (Ministry of Health: 629/2017) and complied with the NIH Guide for the Care and Use of Laboratory Animals.

Muscle acute injury was performed in WT C57BL/6 mice, anesthetized by 1–4% L/min O2 isofluorane (cat.#502017, MWI Vet Supply) inhalation, by inserting a 30-gauge 5/16” needle previously cooled in liquid nitrogen into tibialis muscle for 10 min.

### 4.3. Cell Preparation and Isolation by FACS

Tibialis anterior of mice were subjected to homogenization and enzymatic dissociation according to Marinkovic et al., 2019 [17,29]. The cell suspension was filtered through a 40 μm nylon filter and incubated with the following antibodies for 30 min on ice: CD45 (Invitrogen, 48-0451-82), CD31 (Invitrogen, 48-0311-82), TER119 (Invitrogen, 48-5921-82), SCA 1 (Invitrogen, 11-5981-82), and ITGA7 (AbLab, R2F2). Cell sorting was performed on a DAKO-Cytomation MoFlo High-Speed Sorter (Glostrup, Denmark). Satellite cells were isolated as TER119−CD45−CD31− SCA-1− ITGA7+ and processed at time 0 in lysis buffer to obtain protein extract.

### 4.4. Western Blot Analysis

Tissues were lysed in 50 mM Tris HCl pH 7.5, 50 mM NaCl, 320 mM sucrose, 1% Triton X-100, 10% glycerol supplemented with protease, and a phosphatase inhibitor cocktail. Cells were rinsed in ice-cold PBS and collected in lysis buffer containing 20 mM Tris–HCl pH 7.4, 150 mM NaCl, and 1% Triton X-100 with a protease inhibitor cocktail. Nuclear and cytosolic extracts were obtained using the NE-PER Nuclear and Cytoplasmic Extraction Kit (Thermo Scientific, UK). Protein concentrations were determined by the Bradford assay, using bovine serum albumin as a standard. Aliquots of total protein extracts from cells after different treatments were resolved on SDS–polyacrylamide gel and transferred to a nitrocellulose membrane. Blots were blocked in 5% non-fat dry milk in T-PBS (PBS + 0.05% Tween-20) for 1 h at room temperature and then incubated overnight with the described antibodies. The membranes were incubated with HRP-conjugated secondary antibody for 1 h at room temperature, and the signal was detected by Immobilon^®^ Western (Millipore, Germany).

### 4.5. Antibodies

Anti-tubulin (T-4026, Sigma), anti actin (A2066, Sigma), anti GAPDH (G9545, Sigma), anti RTN-1C (Ab8961, AbCam), anti MyHC (DSHB), anti myogenin (DSHB), anti GRP78 (NBP1-06274, Novus Biologicals), and anti p-eIF2a (ab32157, AbCam) were used.

### 4.6. Immunofluorescence

C2C12 cells were fixed in 2% PFA over night at 4 °C, and then were permeabilized with PBS plus Triton™ X-100 (Sigma, Darmstadt, Germany) 0.3% *w/v* (permeabilization solution) for one hour at RT and then incubated with a blocking solution composed of PBS plus Triton™ X-100 0.1% *w/v*, glycine 1% *w/v* and goat serum NGS (Natural goat serum) 10% *w/v* for one hour at RT. Subsequently, the sections were incubated with a primary antibody appropriately diluted in blocking solution for one hour at RT. Two washes were performed, respectively at 15 and 5 min each, with PBS plus Triton™ X-100 0.2% *w/v* BSA (Bovine Serum Albumin) 1% *w/v* (washing solution), and then the sections were incubated for one hour at RT with a secondary antibody conjugated with a fluorophore appropriately diluted in blocking solution. After a 10 min rinse with the washing solution, DAPI was added (4′,6-diamidino-2-phenylindole), 0.1 mg/mL, in PBS for 10 min at RT. Finally, the sections were mounted with Aqua Poly/Mount (Polysciences, Warrington, PA, USA).

Primary antibody used:

- Mouse anti-MyHC (produced in our laboratory by hybridoma cells) diluted 1:2.

The secondary antibody used:

- Anti-mouse Alexa555 (Molecular Probes) diluted 1:100.

The sections were photographed with the microscope Nikon Eclipse TE 2000, supported by MetaMorph^®^ software (Molecular Devices, San Jose, CA, USA).

### 4.7. Quantitative-RT PCR

Tissues were lysed in Trizol reagent (Invitrogen, Renfrew, UK) and total RNA was extracted using Direct-Zol™ RNA MiniPrep Plus according to the manufacturer’s instructions. One μg of RNA was retro-transcribed using SensiFAST™ cDNA Synthesis Kit (Bioline, Taunton, MA, USA) and used in the quantitative RT-PCR (qPCR) experiment, using SensiFAST™ SYBR Hi-ROX Kit (Bioline, Taunton, MA, USA) following the manufacturer’s instructions. Thermocycling consisted of an initial polymerase activation step at 98 °C for 5 min, and amplification was performed with 35 cycles of 95 °C for 15 s, 68 °C for 10 s, and 72 °C for 20 s with data acquisition at this stage, and the reaction finished by the built-in melt curve. The relative amounts of mRNA were calculated by using the comparative Ct method.

RTN-1C: 5′-TACCCGTCTCCAGGGTAGC-3′, 5′-TTGGTCAATCTGTGCCTGGT -3′

GAPDH: 5′-AACTTTGGCATTGTGGAAGG-3′, 5′-CACATTGGGGGTAGGAACAC-3′

### 4.8. Statistical Analysis

Graph Pad was used for statistical analysis. ImageJ64 software was used for densitometric analysis. Statistical significance was determined using the Student’s *t*-test or one-way ANOVA test. *p*-value smaller than 0.05 (*p* < 0.05) was considered to be significant.

## Data Availability

No new data were created or analyzed in this study. Data sharing is not applicable to this article.
